# The Viability of Photocatalysis for Air Purification

**DOI:** 10.3390/molecules20011319

**Published:** 2015-01-14

**Authors:** Stephen O. Hay, Timothy Obee, Zhu Luo, Ting Jiang, Yongtao Meng, Junkai He, Steven C. Murphy, Steven Suib

**Affiliations:** 1 United Technologies Research Center (ret.), 35 Weigel Valley Drive, Tolland, CT 06082, USA; 2 United Technologies Research Center (ret.), 351 Foster Street, South Windsor, CT 06074, USA; E-Mail: tnobee@snet.net; 3 Institute of Materials Science, University of Connecticut, U-3060, 91 North Eagleville Road, Storrs, CT 06269-3060, USA; E-Mails: luozhu711@gmail.com (Z.L.); junkai.he@uconn.edu (J.H.); steven.suib@uconn.edu (S.S.); 4 Department of Chemical and Bimolecular Engineering, University of Connecticut, U-3222, 191 Auditorium Road, Storrs, CT 06269-3060, USA; E-Mail: tij11001@engr.uconn.edu; 5 Department of Chemistry, University of Connecticut, U-3060, 55 North Eagleville Road, Storrs, CT 06269-3060, USA; E-Mails: yongtao.meng@gmail.com (Y.M.); steven.c.murphy@uconn.edu (S.C.M.); 6 Department of Chemical Engineering, University of Connecticut, U-3060, 91 North Eagleville Road, Storrs, CT 06269-3060, USA

**Keywords:** photocatalysis, air purification, prototype modeling, photoreactor, coadsorption, indoor air, catalyst deactivation, byproducts, catalyst lifetime

## Abstract

Photocatalytic oxidation (PCO) air purification technology is reviewed based on the decades of research conducted by the United Technologies Research Center (UTRC) and their external colleagues. UTRC conducted basic research on the reaction rates of various volatile organic compounds (VOCs). The knowledge gained allowed validation of 1D and 3D prototype reactor models that guided further purifier development. Colleagues worldwide validated purifier prototypes in simulated realistic indoor environments. Prototype products were deployed in office environments both in the United States and France. As a result of these validation studies, it was discovered that both catalyst lifetime and byproduct formation are barriers to implementing this technology. Research is ongoing at the University of Connecticut that is applicable to extending catalyst lifetime, increasing catalyst efficiency and extending activation wavelength from the ultraviolet to the visible wavelengths. It is critical that catalyst lifetime is extended to realize cost effective implementation of PCO air purification.

## 1. Introduction

Using light to achieve clean air and water resources through photocatalytic oxidation is a goal of scientists worldwide [1,2,3]. Success depends on the air or water stream to be purified [4,5,6,7]. Our focus is on the challenges of purifying an air medium, primarily indoor air. United Technologies Research Center (UTRC) devoted significant resources towards this goal over the last two decades. Air itself is a mixed media that may contain a variety of both particulate and gaseous components. Photocatalysis is a widely generic term that applies to chemical change enabled by photon activated catalysis. The chemical change is usually oxidation, but in some cases reduction can be effected. The catalyst is generally a metal oxide semiconductor, usually titania, with an appropriate band gap energy that allows adsorption of an ultra-violet photon to generate electron hole pairs which initiate the chemical change. Generically:

hν + TiO_2_(s) → TiO_2_(s) + h^+^ + e^−^ (1)

For titania the band gap is centered near 360 nm. In air saturated with water vapor and under ambient light, water vapor has chemically adsorbed creating a partially hydroxylated surface. With this in mind we sometimes express Equation (1) as:

hν + TiO_2_^.^H_2_O(s) (sTiO_2_(s) + OH + H (2)

This allows us to think about the chemical change in terms of hydroxyl or proton attack on an adsorbed species and this can be useful in understanding and discussing the chemical changes initiated by photocatalysis. The surface chemistry is extremely rich and complex and depends on morphology of the bulk and surface, and specifically on the termination of the semiconductor surface bond and the myriad species that can adsorb on the surface.

In evaluating the effect of a photocatalytic oxidation (PCO) based air purifier we only need understand what goes in and what comes out in relation to our goal. It is critical that one completely understands the medium to be purified, the resultant fluid, and the desired outcome. If we are talking about polluted ground water the desired outcome is simply to remove the pollutant by chemical change to benign products. In this case we are working with a well-defined system and we need only understand the surface adsorption phenomenon and photocatalytic reactions of a few species. For example, if we look at ground water contaminated with chlorinated solvents from a dormant degreasing pit, then the solvents most commonly used were TCE and PCE. The gas over the contaminated ground water would consist of water saturated air, contaminated with TCE and/or PCE, and we look to construct an air purifier that removes the contaminants [7] or chemically changes them to benign or easily removable products. This system is well defined, has a fixed set of species to oxidize with a slowly varying source rate, and serves to define one limit of the variety of uses for an air purifier.

Another extreme occurs when evaluating the effect of a catalytic air purifier on indoor air. In this case the challenge fluid is complex and the goal is either to effect a change to a healthier environment, without impacting comfort, or to reduce outdoor air intake while maintaining air quality and comfort. The latter allows energy savings to be realized by minimizing conditioning (heating and cooling). In some cases, the size of the Heating, Ventilation and Air Conditioning (HVAC) equipment may be reduced resulting in capital savings on equipment. The components of indoor air that affect the human condition are myriad and both particulate and gaseous. Within the set of all particles, ultra-fine particles have been directly linked to heart health [8]. Bioaerosols can be allergens, asthmatic triggers, or mold spores [9], and some particles are benign. Within the set of gaseous products some are carcinogens; some cause respiratory distress; some are toxic; some are odiferous and some are benign. If we wish to treat indoor air to make it “healthy”, one technology alone will not suffice to treat the wide range of particulates that may be encountered, as well as the wide range of gaseous components.

In fact, the stated goal of creating a healthy environment is itself nebulous. Individuals respond differently to different exposures [8]. In order to comment on either the efficiency or validity of an air purifier in this case, we first need to understand the challenge. Therefore, we need to understand indoor air and its components, we need to understand how the mixture of species adsorbs on the catalyst surface. We need to understand how this mixture reacts in an Ultra-Violet Photocatalytic Oxidation (UVPCO) air purifier and what is contained in the resultant mixture of effluents. In addition we need to understand this both initially and on a steady-state basis since UVPCO air purifiers will not have total mineralization capacity for all species and may produce hazardous by-products. In other words, transient effects may be important both as the air purifier reaches steady-state operation and as the building environment changes diurnally and seasonally.

To accomplish this we will look closely at indoor air and its components. We will restrict the photocatalyst to titania and modified titania. We will examine what occurs to those components when passing through a photocatalytic reactor and review the approach taken by UTRC in the construction of an UVPCO air purifier and validation of its effect in a real indoor environment.

## 2. Results and Discussion

The challenge in defining indoor air is that it is a mixed medium with variable components [10]. Indoor air is a mixture of outdoor air and recycled indoor air that may have been conditioned. Outdoor air comes from either or both ventilation and infiltration and is highly dependent on the quality of the outdoor air. Recycled air has been contaminated with a plethora of sources including construction materials, furnishings and occupants. In reality, each building whether an individual home or high-rise office building presents a unique and time-varying challenge, but if we limit ourselves to certain typical types of buildings in typical places, we can begin to define indoor air sufficiently to understand the operation of a photocatalytic air purifier.

### 2.1. Indoor Air

As stated, indoor air consists of both aerosols and gaseous components. Aerosols can be mineral, liquid or biological. In general, aerosols are either removed from indoor air by filtration or fine particles remain suspended in air. In a typical office building environment, large particles are filtered by low pressure drop filtration on the air intake and in the recycled flow during conditioning. Some bioaerosols have been demonstrated to be oxidized by photocatalysts, but, in general, this causes either reversible or irreversible deactivation of the photocatalyst and is undesirable. For this reason a higher quality filter is placed in the air stream prior to the catalyst, at the expense of an increase in pressure drop. The exact specifications of the filter are dependent on the design of the HVAC system in which the purification unit is located, specifically the pressure drop tolerance, the air flow rate and the dimensions of the photocatalytic unit. The goal is simply to insure minimal contact of aerosols with the surface of the photocatalyst. Particles will either be trapped by the filter or fly over the surface of the photocatalyst without impingement. A useful target is the highest efficiency filter allowed by pressure drop constraint. This implies that we can largely ignore the aerosols in indoor air and focus on the effect on the photocatalyst on the gaseous components.

The US EPA has commissioned the Building Assessment Survey and Evaluation (BASE) study [11] to determine the gaseous components in indoor air in 100 randomly selected office buildings in the United States. Using standardized protocols they collected extensive data in 37 cities in 25 states. The preliminary results of this study are partially summarized in the first two columns of Table 1. 

**Table 1:** Selected VOCs found in the US EPA BASE study and their contribution to UTRC’s proposed tolerance metric.

Compound	Average Concentration (ppbv)	Contribution to Tolerance Index	Origin
*Formaldehyde*	11.6	0.387	TLV
*Acetaldehyde*	4.2	0.0627	OT
*Benzene*	2.1	0.0339	SMAC
*Toluene*	8.3	0.00519	OT
*1,4-Dichlorobebzene*	1.4	0.0117	OT
*Carbon disulfide*	1.1	0.0115	OT
*Styrene*	1.6	0.0114	OT
*Butyl acetate*	3.3	0.0106	OT
*m,p-Xylenes*	3.3	0.00243	OT
*Decane*	1.4	0.00190	OT
*Undecane*	2.0	0.00167	OT
*Acetone*	27.1	0.00131	SMAC
*Dodecane*	2.0	0.0010	OT
*Naphthalene*	13.0	0.342	OT

In normal indoor air, there are *ca*. 200 individual gaseous components, most in the 10 ppb range or lower, and most are volatile organic compounds (VOCs). The average tolerance index of the air found in office buildings by the BASE study is 0.884. In problem indoor air, air that has generated complaints and or illness, there may be a considerably higher total or higher concentrations of individual components, resulting in a significantly higher tolerance index.

If our goal is to change air quality, we can simply rate the effect of an air purifier based on its efficiency. However, in treating indoor air our goal is to create cleaner or healthier air. This goal is somewhat nebulous as there are different effects that can be exhibited by different VOCs. Some VOCs such as formaldehyde and benzene are carcinogens, some are toxic, some are odorous and some are benign. In order to quantify the effect of a photocatalytic air purifier one needs a metric to describe the ability of humans to tolerate an environment with any given mixture of contaminants. One such metric is proposed by Hollick and Sangiovanni [12]. Based on existing metrics for allowed VOCs in Spacecraft, odor thresholds, toxicity levels or exposure limits, the proposed tolerance metric defines a yardstick for measuring the tolerance of human beings to a given indoor air mixture. This metric also allows us to model the effect of a photocatalytic air purifier on the building environment. They define a Tolerance Index, T_i_, for each VOC component in indoor air as follows:

T_i_ = C_i_/A_i_ (3)

where C_i_ is the actual concentration of contaminant i and A_i_ is the maximum acceptable concentration for each contaminant. The metrics used were derived from three factors:

1 NASA’s Spacecraft Maximum Allowed Concentration (SMAC) levels
2 One tenth of ACGIH’s Threshold Limit Values (TLV) levels
3 Odor Thresholds (OT)

Multiple limits are used since no consensus exists in the indoor air scientific community as to what constitutes a healthy indoor environment and what measurable metric can be used to assess indoor air. Most species are limited by their odor threshold. TLVs are eight hour exposure limits over a worker’s lifetime. One tenth of the TLV value was taken to insure a conservative metric for air exposure which can occur on a twenty four hour basis.

Air quality is assessed by summing the individual contributions to the tolerance matrix:

ƩTi < 1.0 indicates acceptable VOC concentrations
(4)

The top fifteen contributors to the tolerance metric culled from the EPA Base Study are shown in the latter two columns of Table 1. The dominant effect on air quality, based on this metric, is due to formaldehyde (limited by TLV) and napthalene (limited by odor). The use of a metric such as this allows validation of the operation of a UVPCO air purifier on complex media, such as indoor air. Other similar metrics have been proposed, but, so far, none have gained wide acceptance and use.

### 2.2. Design of an Air Purifier

A photocatalytic air purifier uses a catalyst, a substrate to support the catalyst in the air flow, and a light source. In addition, the purifier must be housed in an accessible and safe fashion, while integrating the device into the air supply it was designed to act upon.

The design [13,14,15,16,17] of a PCO air-purifier balances efficiency and cost. The capital cost, alone, is due to the housing, the light source and the catalyst/substrate. Cost is spread about equally across these three categories. For indoor air purification the cost of operation consists of the electricity used in the lamps, and ballasts, if fluorescent lamps are used. Maintenance costs include lamp, ballasts and catalyst replacement. Catalyst lifetime dictates its replacement schedule. If the device is placed in a flowing airstream provided by an existing ventilation system, the design must also consider the allowable pressure drop. If a fan is used, that cost is added to the initial and maintenance costs. If a filter is used to protect the lamps and or the catalyst, filter replacement cost is added to the maintenance cost, and if the filter is high efficiency, the filterdrop must be considered.

**Figure 1:**
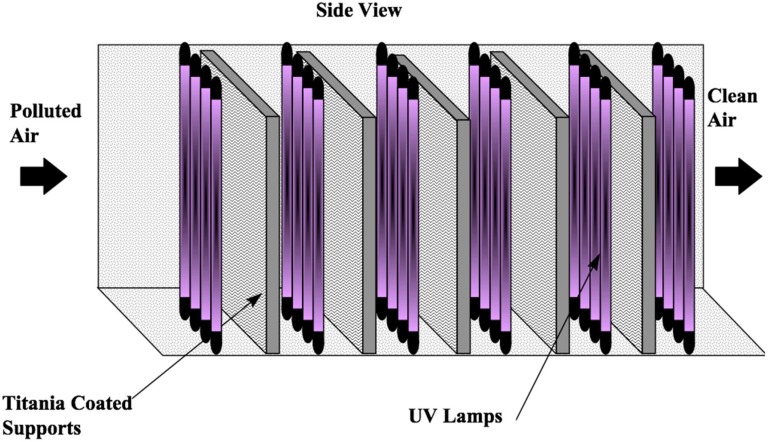
Generic Multi-stage, Honeycomb-Monolith Photocatalytic Reactor.

The standard catalyst for many photocatalytic applications is Degussa P-25. P-25 is cost effective, readily available and exhibits high photocatalytic activity for many species of interest in indoor air. The catalyst support needs to provide a large surface area in contact with the flowing air stream, a low pressure drop and structural integrity. The support should also lend itself to ease of manufacture and have sufficient integrity to survive shipping and handling. Ease of prototyping and the ability to easily modify the design for size are also considerations. Based on these considerations, UTRC uses a modular approach to the purifier design utilizing aluminum honeycomb as a catalyst substrate. The basic module is a light source and a titania catalyst supported on the honeycomb. A PCO air purifier of variable efficiency (see Figure 1) can be constructed by using multiples of this module and this can be designed to fit pre-existing spatial constraints.

Titania is activated by photons with energy greater than the band gap (*ca*. 360 nm.) Light sources (see Table 2) may be fluorescent specialty lamps, LEDs or any other photon emitter having the required wavelength. The Sun is free, but light is hard to deliver where needed, and is only available during daylight hours. The cheapest, longest lifetime and most readily available light sources are UV fluorescent lamps. UTRC uses fluorescent lamps in their modular design. LED sources need lower wavelengths and longer lives to be a viable alternative. UV fluorescent lamps are based on the mercury vapor spectra. Germicidal lamps emit principally at 254 nm. Black light fluorescent lamps are coated with a manufacturer-dependent phosphor. This causes slight variations in emission spectra, but are generally centered near 360 nm and have a *ca*. 50 nm FWHM bandwidth. Lifetimes are approximate and both manufacturer and mode-of-operation dependent.

In rate measurements performed in a flat plate reactor [18] which will be described later in greater detail, we see no measurable difference in photocatalytic (precursor disappearance) rates obtained with germicidal lamps and those obtained employing black light lamps. This is attributed to the tradeoff that exists between the black light source where the emission band overlaps the titania band gap adsorbing *ca*. 70% of the emitted photons and the *ca*. 70% fewer photons per watt generated at 254 nm. Photocatalysis is a photon initiated process, and the small differences between the number of photons absorbed per Watt at these two wavelengths is ameliorated by the *ca*. 0.6 power intensity dependence observed at the intensities used for rate measurements and purifier design. Therefore, the sole considerations for fluorescent lamp choice are cost and the outcome desired. Indoor air contains bioaerosols both viable, such as mold spores, bacteria and airborne viruses and nonviable, such as allergens. Germicidal lamps can inactivate viable bioaerosols as they fly through the irradiant field. If this effect is desired, then germicidal lamps are the lamp of choice and the housing must be designed to be resistant to damage by UV light. Black light wavelengths are not strongly germicidal and are more material friendly. In either case, bioaerosols can settle on the photocatalyst and cause deactivation, blocking the surface until they are mineralized. If bioaerosols are mineralized to non-volatile compounds, the deactivating effect may be permanent. Most bioaerosols are either captured by a filter or fly past the catalyst surface entrained in the airstream.

**Table 2:** Common UV light sources for a UVPCO air purifier.

Photon Emitter	UV Wavelength Range	Approximate Lifetime
Sun	UVA and UVB; most UVC is adsorbed in the atmosphere	exists with daylight
Black Light Fluorescent	UVA (260 nm ± 50 nm FWHM)	5000–12,000 h, usually limited by phosphor degradation
Germicidal Fluorescent	UVC (254 nm)	10,000–20,000 h
LEDs	Various, 190 nm to 1100 nm	wavelength dependent, a few thousand hours at short wavelengths

The housing should have easy access to the UV bulb, catalyst/substrate and filter replacement and should fit in a building airstream, preferably downstream of the HVAC unit. The housing also must be sized appropriately for the space available. All interior surfaces should reflect the wavelengths used to excite the photocatalyst. If UVC excitation is used, all parts exposed to UVC radiation should be resistant to UV degradation.

It is cost prohibitive to design an air purifier to be 100% efficient. In most cases a design goal of *ca*. 10%–20% single-pass removal efficiency for formaldehyde is achievable and will result in cleaner air through recycling. An effective clean air delivery rate (CADReff), which is the preferred design parameter rather than single-pass efficiency, is calculated based on the mole fraction (X) of individual contaminants in the air, the reactors single pass efficiency (SPE) and the air flow rate:

CADReff = (SPE)(Flow rate) = X_1_CADR_1_ + X_2_CADR_2_ + … (5)

**Figure 2:**
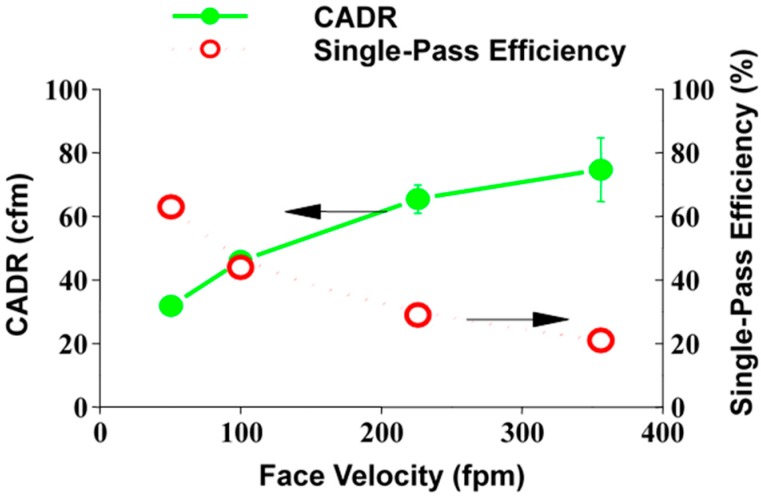
The effect of flow velocity on the effective CADR is opposite to the effect on SPE and gives a more accurate picture of the effect of air purification to HVAC professionals. A generic HVAC design is assumed.

The effective CADR increases with increasing number of modules, and with flow rate, as shown in Figure 2. Using an effective CADR allows us to compare the effect of a photocatalytic air purifier with ventilation. ASHRE requires outdoor ventilation of 15 CFM per person unless air purification is used. If used, verification is required that indoor air quality is maintained. Using an effective CADR allows comparison of the cost of ventilation (heating and cooling) with the cost of air purification.

This is the basic modular design used in a series of prototypes and products deployed by United Technologies. UTRCs steps for the design and validation of a UVPCO air purifier are:

1. Measure reaction rates as a function of humidity and contaminant concentration.
2. Understand the effect on rates due to mixture of contaminants
3. Model and validate the effect of prototype air purifiers
4. Validate prototypes in indoor air
5. Design and validate products

The model was used to design the prototypes which, in turn, were used to validate the model. As will be shown, external validation was also effected through cooperation with the University of Arizona, Harvard University, Danish Technical University, the University of Wisconsin, the University of Connecticut, the University of Nottingham, Lawrence Berkeley National Laboratory and others. Some results remain unpublished and unavailable for review.

### 2.3. Reaction Rate Studies

A complete set of intrinsic rate data, assembled as outlined above, serves as essential input to a design procedure for a photocatalytic air-purifier.

The rate (R) for photocatalytic oxidation of a contaminant species (X) over TiO_2_ can be expressed as:

R = k_obs_ I^n^ [X_(s)_]^m^ [H_2_O_(s)_] [O_2(s)_] = k'_obs_ [X_(s)_]^m^, where (6)

k'_obs_ = k_obs_ I^n^ [H_2_O_(s)_] [O_2(s)_] (7)

In other words, at constant UV intensity and constant water vapor and oxygen concentrations the rate is proportional to the surface coverage of the species X. At low concentrations the rate (R) is linear with respect to the contaminant X, so we may express the relation as:

R_x_ α [X_(s)_] = S_x_ (8)

where the rate of disappearance of species X is proportional to the surface concentration of X. The photocatalysis of gaseous species can be viewed as a multi-step process where adsorption of gaseous species onto the catalyst surface occurs first. All the interesting chemistry in this process occurs at the gas-solid interface between the photocatalyst, for example, solid titanium dioxide (TiO_2_), and a contaminated airstream. A basic description of the process is accomplished by separating the varied chemical and physical processes that occur into four different categories:

1. Coadsorption of the gas phase species on the semiconductor surface. This includes water, oxygen molecules, the species to be oxidized, and any other species present in the gas phase that compete for surface sites.
2. Activation of the semiconductor surface by a UV photon, generation of electron-hole pairs, followed by the competing processes of recombination and trapping. The trapping species are generally believed to be surface oxygen and water respectively resulting in hydroxyl (OH) and superoxide (O_2_^−^) radicles
3. Initiation, where the free radicals produced by trapping the electron-hole pair initiate attack on the species to be oxidized. This step removes the precursor and the rate of removal is the rate generally measured.
4. Propagation, where sequential free radical attack causes degradation of the reactant species to products and, in some cases, stable by-products. Deactivation of the catalyst, either reversible or irreversible can occur during this step. Intermediate free radicals can bond to the catalyst surface or non-volatile products can form.

The solid titania surface, in air and ambient light, is an active surface, in which water has chemisorbed forming ca. one third hydroxyl terminal groups. Molecular physical adsorption from the gas phase is dominated by the strongest force, *i.e*., by the largest molecule-to-surface-site binding energy. For small molecules the dominant intermolecular forces are hydrogen bonding, dipole-dipole interactions and London forces. One of the earliest published studies of this effect is Obee and Hay [19] and their results show marked dependence on surface binding energy. In brief, they demonstrate that organic molecular functionality and the resultant hierarchy of intermolecular forces (IMFs) dictated the relative reaction rate. 1-Butanol is shown to have a larger rate of photocatalytic removal than 2-butanone, which is larger than 1-butene, which is larger than *n*-butane.

One way that the adsorption of molecules on a surface can be expressed mathematically is to relate the surface concentration S_i_ to the collision frequency of the molecules with the surface, pr and the retention time, ncy of the molecules with the surface:

S_i_ = Zτ = <v> n_i_ στ (9)

The collision frequency can be expressed as a product of the average molecular velocity <v>, the gas phase number density n_i_, and the collision cross-section σ. The average molecular velocity can be expressed in turn as:

<v> = {8kT/πm}^1/2^ (10)

where T = temperature and m = mass of the species. The average time spent on the surface, τ, can be expressed as:

τ = τ_0_e^Q/kT^ (11)

where q_0_ is a constant, and Q is the binding energy to surface. If we insert these expressions into Equation (11) we obtain:

S_i_ = {8kT/πm}^1/2^nσ τ_0_e^Q/kT^ (12)

Equation (12) tells us that surface coverage S_i_ depends directly on the gas phase number density or concentration and on the molecular mass, the binding energy to the surface and the bulk temperature. What we expect is a photocatalytic removal rate that depends on light intensity and surface species coverage. If light intensity, concentration, and temperature are kept constant, and the variation in molecular mass is small, surface coverage will depend on binding energy as shown by 1-butanol, 2-butanone, 1-butene and *n*-butane. The inverse square root dependence of the surface coverage to the molecular mass is slightly misleading since the binding energy to the surface also depends on molecular mass. This is also illustrated by Obee and Hay [19], who performed a series of rate measurements with the straight chain alkanes, *n*-butane, *n*-hexane and *n*-decane. In the limit of other binding energies being approximately equal the molecular size effect dominates. The larger molecule exhibits the largest Van der Walls effect and the largest observed removal rate. The rate of mineralization is distinctly different. Acting conversely to the above effect, mineralization reaction sequences require additional radical attack or addition steps to mineralize larger molecules.

Indoor air is predominantly composed of N_2_, O_2_, H_2_O and CO_2_ with trace contaminants as discussed above. Of these major components only water binds strongly to the hydroxylated titania surface. It is water therefore that is the major adsorbent on the titania surface. All trace contaminants that we wish to oxidize must compete with water adsorption. This competition affects their disappearance rate.

#### 2.3.1. The Effect of Concentration on Rate and the Extent of Mineralization

**Figure 3:**
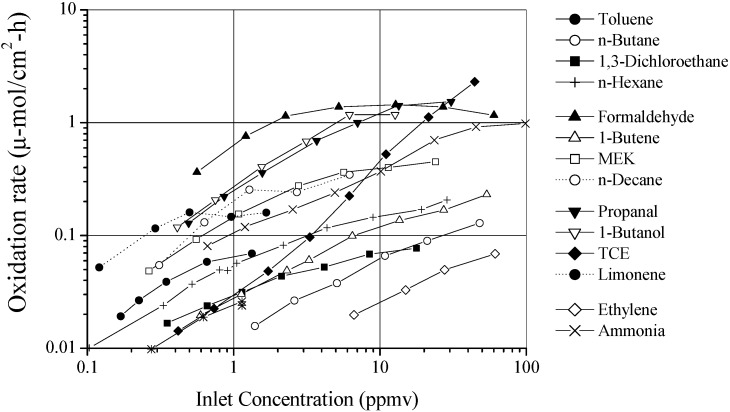
Measured PCO reaction rates for VOCs of interest in indoor air; UV 1-mW/cm^2^, 6000 ppm water level.

In a well-conditioned building (20% to 60% RH at 20 °C) water concentration is in the 6000 ppmv to 16,000 ppmv range. In order to observe the effect of contaminant concentration on removal rate we must fix water concentration and light intensity and wavelength to achieve the relation given in Equation (10). At the drier end of building air (6000 ppmv of water) we minimize the effect of multiple water layers covering the surface of the titania. Figure 3 shows the removal rate for 14 common air contaminates over a range of concentrations from 0.10 to 100 ppmv. Light Intensity was fixed at 1 W/cm^2^ using standard UVC germicidal fluorescent lamps and water concentration was kept standard at 6000 ppmv. At lower concentrations (<0.50 ppmv) the curve of oxidation rate *vs.* gas-phase concentration is near linear, at higher concentrations the curve appears to roll off or stabilize, while at still higher concentrations some species rates decrease while other species increase. Various competing effects contribute to these observations; TCE, for example, can dissociate under UV irradiation.

**Figure 4:**
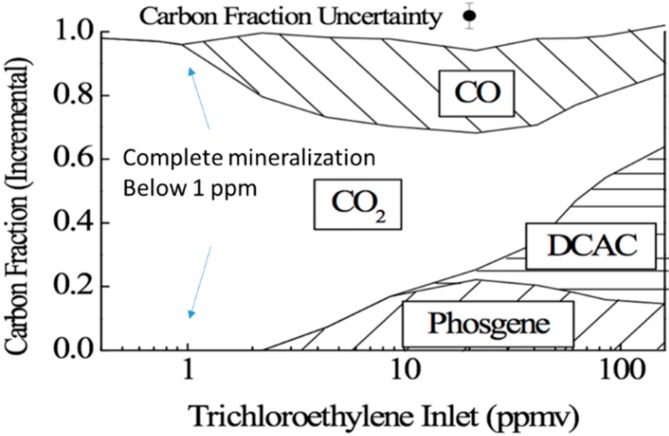
Measured PCO products vary with increasing concentration. Complete mineralization occurs at low concentrations. A different version of this diagram is shown in ref. [7].

As concentration increases, some species saturate the surface, some form additional layers of secondarily adsorbed species, some intermediates chemisorb, blocking active sites, and some form gas-phase free radicals by radical metathesis. Each contaminant behaves uniquely in its competition with water and other contaminant molecules to adsorb (and react) on titania. Hay and Obee [7] showed a map of the product space for TCE as a function of concentration. Light intensity, water vapor concentration and oxygen content are held constant, and they look at the products formed when TCE is photo oxidized over titania (Degussa P-25). The carbon in TCE mineralizes completely to CO_2_ at concentrations less than 1 ppmv. Over this limit the carbon fraction of CO_2_ decreases, the CO carbon fraction increases from *ca*. 0.0 at 1.0 ppmv TCE to *ca*. 0.70 at *ca.* 20 ppmv TCE. Phosgene (COCl_2_) appears as a product over 1.1 ppmv TCE and increases with TCE concentration to ca. 11 ppmv TCE, then decreases with increasing TCE concentration. Dichloroacetyl chloride begins to appear at *ca*. 10 ppmv TCE and its carbon fraction rises with increasing TCE concentration (see Figure 4). In the limit of low concentration, when the number of active sites dominates the number of adsorbed contaminant molecules, complete mineralization will occur. The exact range of concentrations for this limit to be observed depends on light intensity, the nature of the photocatalyst and the contaminant molecule. At higher concentrations incomplete mineralization results in byproducts. Hay and Obee observed stable molecular byproducts that desorb from the surface reentering the gas stream. Other more transient byproducts exist but do not survive long enough to be detectible in the gas phase. This is a critical observation for treating indoor air quality, where most contaminants are in the ≤ 10 ppbv range. We expect complete mineralization of contaminant molecules unless the contaminant exists at high concentration due to a spill or specific high contaminant emission source. They also observe gas phase oxidation of TCE and PCE in the absence of a photocatalyst. Photodisociation can occur at the wavelengths in use. This effect dominates at high concentrations.

#### 2.3.2. The Effect of Humidity on Rate

As previously discussed, water is the major adsorbent on the hydroxylated titania surface and, as such, all other adsorbed species compete with water to adsorb. Species with a strong affinity to the surface compete with a higher degree of success. Obee [18] showed this effect of humidity on the photooxidation rate of toluene and formaldehyde, which possess a weak and a strong molecular dipole moment respectively. One expects formaldehyde with its strong molecular dipole to compete successfully, and this is illustrated by the data. Formaldehyde oxidizes at a faster rate. At 2.2 ppmv the reaction rate of formaldehyde is faster and increases with increasing humidity to *ca*. 2500 ppmv water, the rate then gradually declines with higher water concentration. If we equate the rates observed to surface coverage this behavior is only partially explained by the competition between water and formaldehyde for surface adsorption sites. Above *ca*. 2500 ppmv water, formaldehyde’s diminishing disappearance rate is explained by increased competition from water due to an increase in the gas phase water concentration. However, in photocatalysis, water has a dual function. H_2_O adsorbs on available sites blocking formaldehyde from adsorbing, and is split by the photo-activated catalyst to produce radicals. In the limit of low concentrations of water and formaldehyde, there is no coadsorption affect and the rate of formaldehyde disappearance increases with increasing formaldehyde concentration. However, here the formaldehyde concentration is kept constant, so the increase in formaldehyde disappearance rate correlates to increasing water concentration. This can be attributed to increased radical production from water and resultant larger turnover rate for formaldehyde on the surface. If this description is accurate, changing the contaminant molecule from formaldehyde to a larger, but more weakly bound molecule, such as toluene, results in a lower surface concentration of contaminant and a resultant lower oxidation rate. At similar gas phase concentrations (2.2 ppmv formaldehyde and 5.4 ppmv toluene) the disappearance rate of toluene is depressed by a factor of *ca.* 10 over the formaldehyde rate.

As water vapor concentration increases to those representing high humidity in a building, both reaction rates reduce, the weaker bound species affected to a greater degree. A bimolecular Langmuir-Hinschelwood rate expression seems to fit the data well, especially at lower water concentration. Obee and Brown [20] studied the effect of water vapor concentration on the same two species and butadiene. In all cases the effect of humidity was to increase the rate as humidity decreases until a limit of increased oxidation is reached. This effect is observed when water is less than 1000 ppmv (approximately 30% RH). These are very dry conditions for indoor air and this effect can be neglected in most case. In a well-conditioned building humidity is maintained between 20% and 60% RH at *ca*. 20 °C, oxidizable contaminants in indoor air will exhibit higher reaction rates at the lower end of the humidity range.

#### 2.3.3. The Effect of Temperature on Rate

Temperature can affect a semiconductor such as titania by promoting thermal catalysis. In controlled buildings the temperature range is small, and this effect, while shown to exist, is negligible. Surface coverage does depend on temperature, which affects the collision frequency of the gas phase molecules with the titania surface, but this is a small effect in the tight range of temperatures manifested by conditioned indoor air. Ordinarily, the expectation is that surface concentration decreases as temperature increases. In some cases, when partitioning across the surface occurs with large differences in binding energies, a different phenomenon dominates and the reaction rate for the more weakly bound species can increase with temperature.

If we restate Equation (12) and hold concentration constant while allowing temperature to vary, surface coverage is proportional to the binding energy, the temperature and the mass of the species. We can express the result as:

S α e^Q/kT^/m^½^ (13)

If we form a ratio of the rate of surface coverages of water to ethylene we find:

S_w_/S_et_ α e(^Q^_w_^−Q^_et_)^/kT^ or e^Q^_w_^/kT^
when ^Q^_e_ << ^Q^_w_ (14)

This shows that the ratio of relative surface coverage depends only on the binding energy difference and the temperature. When the binding energy of ethylene is much less than the binding energy of water, this ratio will decrease with increasing temperature, and so as the ratio of surface coverage of water to that of ethylene increases, so will the ratio of measured rates. This effect was seen by Obee and Hay [21], over the temperature range 2 °C to 48 °C. They modeled this effect using a temperature dependent form of the Lanqmuir-Hinshelwood rate expression. The photocatalytic removal rate of ethylene is observed to increase with increasing temperature. This effect will be observed for all species with small binding energies. Increasing temperature does not occur to any great extent in buildings, so this effect can be neglected when treating indoor air.

#### 2.3.4. The Effect of Mixtures on Rates

The effects of coadsorption on the titania surface also dictates how a mixture of oxidizable gaseous contaminants will react photocatalytically. In the limit of low humidity and low concentrations of contaminants all species will adsorb on the catalyst surface partitioned solely by the effects of relative concentration and binding energy. In this limit, all oxidizable species react simultaneously. As humidity or total contaminant concentration increases, increasing competition develops for adsorption sites, and as concentrations increase the species with the strongest adsorption binding energy dominates the photocatalytic process. In the extreme of high contaminant concentration, Zorn, *et al.* [22] observed this effect in a recirculating photoreactor system. Sequential reaction occurs based on the strength of bonding to the surface. They studied the compounds ethanol, ethanal, propanone, propene and propane both singly and in mixtures at the 100s of ppmv concentration level. All five individual reaction rates were measured and fell in a sequence dictated by relative strength of bonding to the surface. In mixture of propene and propanone, a degradation of the propene oxidation rate was observed until all propanone was oxidized. In a separate experiment, where ethanol was injected into a photoreactor after propanone is *ca*. 80% removed by PCO, the reaction of the ketone was halted by the alcohol and further disrupted by the aldehyde intermediate observed in ethanal PCO. Only when the alcohol and aldehyde intermediate disappear does the photooxidation of the ketone resume. In indoor air we expect the contaminant mixture to be near the limit of low concentration. Normal indoor air has a total contaminant load that is less than 1 ppmv, usually much less. In sick buildings, or in special circumstances the contaminant load may be higher and in these cases, the effects of coadsorption and byproducts become more significant. This effect is correlated with some success to Henry’s Law both in Zorn, *et al*., and Hodgson, *et al*. [23].

As we have seen with the example of TCE, byproduct formation can depend on parent concentration. This could be related to the ratio of activated surface site or radicals formed to adsorbed species and the number of steps in the mineralization reaction sequence. Byproducts form when mineralization proceeds through sequential free radical attack and a stable molecule forms as an intermediate. If a stable intermediate forms it competes with all other species to be retained on the surface or further oxidized. If this analogy holds, one expects more byproducts at higher total contaminant concentration in a mixture such as indoor air. In indoor air this means that we expect by-product formation to be important if any volatile VOC is introduced into the environment in high quantities. This could be the consequence of a spill or use of a highly volatile solvent during maintenance. As in the experiment conducted by Zorn, *et al.*, a byproduct will be oxidized further in time by a recycling system, but understanding the potential for such events is critical.

**Figure 5:**
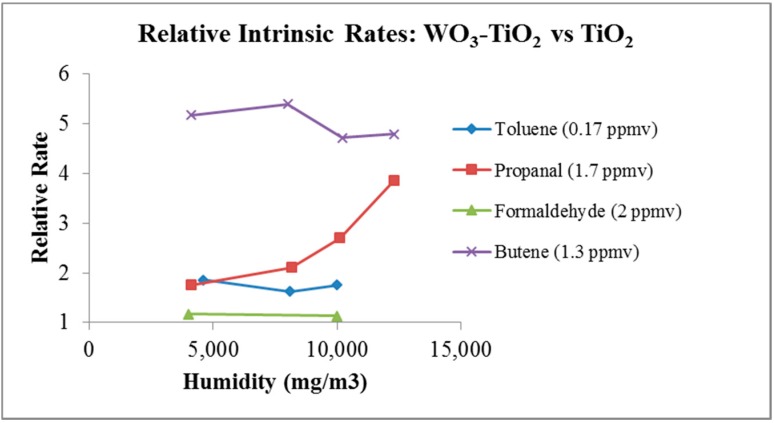
A partial surface layer of WO_3_ modifies the catalyst surface, this changes the binding energies in such a manner that VOC adsorption can compete with water adsorption on the catalyst surface. The relative rate is the PCO removal rate observed for (WO_3_-TiO_2_) minus the PCO removal rate for (TiO_2_) divided by the removal rate for (TiO_2_).

If the surface morphology of the catalyst is altered, the coadsorption phenomenon may be altered. Wei *et al*., patented [24,25] a method of creating a 3% WO_3_ coating on Degussa P-25. This modifies the surface from the hydrated titania surface to one that is partially hydrated titania and partially WO_3_. The photochemical removal rate is enhanced for most VOCs, formaldehyde excluded, by this modification. Enhanced photocatalytic efficiency is shown for propanal, toluene and butene. High efficiency also ameliorates the effect of humidity by lessoning the effect of water coadsorption (see Figure 5). This manifests in higher removal rates for these species at high water vapor concentrations. This is attributed to the surface modification where water does not hydrogen-bond to WO_3_ as it would to the hydroxylated titania. This allows most VOCs more efficient competition for adsorbent site. In indoor air applications, surface modification of titania can enhance overall purifier efficiency.

#### 2.3.5. Deactivation

Deactivation of the titania photocatalyst surface can occur by either reversible or irreversible means [26]. When a VOC containing carbon, oxygen and hydrogen is oxidized by the hydroxyl or superoxide radical to complete mineralization, the products are carbon dioxide and water. This regenerates the partially hydroxylated titania surface. Radicals formed during the mineralization reaction sequence may chemisorb on the surface. This can occur in a reversible fashion. If the chemisorbed species can oxidize further by radical attack to carbon dioxide and water the original surface regenerates itself. This regeneration may also be effected by calcining. In practice a photocatalytic air purifier may suffer reversible deactivation due to periodic fluctuations in VOC concentration. Operation during times when the building is devoid of occupants, which is associated with low total contamination rates, can serve to regenerate the catalyst. Irreversible deactivation can also occur if a non oxidizable species forms on the surface permanently blocking previously active sites or denying access for adsorption. Cao, *et al.* [27] studied toluene oxidation at high (relative to indoor air) concentration (10 ppm) concentrations using various forms of titania and platinized titania. The study is performed using UTRCs flat plate reactor, as described above, to measure rates. They contrast the performance of Degussa P-25 with three nanoscale titania materials, one partially platinized. The three nanoscale materials were prepared by sol-gel methodology and differed either in calking temperature (350 °C or 420 °C) or the addition of 0.5% Pt (350 °C). The nanoscale titania was found to be more reactive to toluene that P-25, but rapid deactivation was observed due to blocking of active sites by the partially oxidized intermediates, benzaldehyde and benzoic acid. The platinized nanoscale titania exhibits a lower reaction rate but deactivates slower. The deactivation was reversed by heating at temperatures above 420 °C. The more active the surface, the faster deactivation can occur.

In the same reactor, Huang, *et al.* [28] studied the photooxidation of tetraethylamine (TEA) over Degussa P-25. The disappearance rate was found to vary with concentration, humidity and light intensity in a manner consistent with that discussed above. The study was conducted with TEA in the concentration range 5.0 ppmv to 20.5 ppmv. Again deactivation is observed. Fourier transform infrared (FTIR) and temperature programmed desorption with a mass spectroscopic detector (TPD-MS) were used to probe the deactivation. They observed a marked concentration dependence on the degradation. At 20.5 ppmv TEA deactivation occurs rapidly and is near total after 90 min., while at 5.0 ppmv some activity remains after ten hours. They also observe that incomplete oxidation (or byproduct formation) increases as deactivation proceeds. If this behavior is universal, then as deactivation proceeds by-product formation could increase. Intermediates are either chemisorbed on the surface, as with TEA, released as stable molecules into the air stream, or adsorbed on the surface and further mineralized.

These varied results yield a snapshot of how an air purifier affects indoor air. We expect the effects to be humidity and temperature dependent. The effect of temperature is minimal in a conditioned environment. Increasing humidity will depress reaction rates but tailoring the surface morphology to lessen water adsorption can ameliorate these effects. At indoor air concentrations, we expect all species to photo-oxidize independently unless there is a spike in the concentration of one or more contaminants. Recycling air will minimize the effect of a momentary increase in VOC concentration, but the effect of by-products (both transient and steady-state) on occupants needs more study.

## 3. Experimental Section

### 3.1. Photocatalytic Reaction Rate Reactor

The main purpose of our photocatalytic flat-plate reactor is to generate intrinsic oxidation rates for selected gaseous species of importance to indoor air quality. Design features include uniform irradiation of the photocatalyst surface, radiation of appropriate range of wavelength, and elimination of mass-transport influence (on the reactants and reaction byproducts) between the flow field and photocatalyst surface. Later these data are fed into an air purifier design procedure that explicitly accounts for non-uniform distribution of radiation on the photocatalyst surface and mass-transfer effects between the flow field and photocatalyst surface.

**Figure 6:**
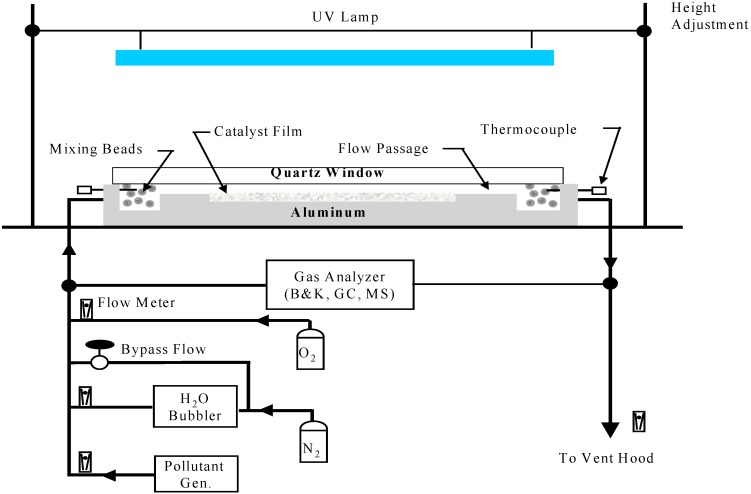
Photo-oxidation Reactor.

Our photocatalytic reactor design is shown in Figure 6. Details of the reactor, detection apparatus, and experimental procedures used to obtain rate data are given elsewhere [18,20]. The reactor design allows for measurements of intrinsic destruction rates free from mass-transport (diffusion) effects, and for study of the effects of contaminant concentration, humidity, and UV intensity-dependencies on their rates of disappearance.

A flow by-pass valve allows complete adjustment of the delivered humidity level. An approximate atmospheric level of oxygen (15%) and nitrogen (85%) closely mimics the targeted application environment (residential and building air). The oxygen level is not critical as long as the level is maintained above about ~1% [2,29], (reaction rates follows Langmuir-Hinshelwood kinetics and is zero order for oxygen levels exceeding ~1%).

Titania-coated glass (or aluminum) plate are placed in a well (25 mm by 46 cm) milled from an aluminum block, and covered by an appropriate quartz window (UV transparent). Gaskets between the quartz window and aluminum block creat a flow passage of 25 mm (width) by 1–2 mm (height) above the titania-coated glass-plates.

In this reactor an opaque film of the photocatalyst, Degussa P-25 titania, is deposited on flat 25 mm wide microscope slides using a wash-coat process. The wash-coat solution is prepared by suspending the titania (5% by weight) in distilled water. The slides are dipped in the wash-coat solution several times, air dried between dipping, and then oven dried at 70 °C. This process is repeated until a sufficient loading (≥0.74 mg/cm^2^ film per side) is achieved.

In a study of film loading by Jacoby [30] the oxidation rate of trichloroethylene increased with film loading up to a P-25 titania loading of 0.5 mg/cm^2^ and remained constant for all higher loadings. This finding suggests that the oxidation rate maximizes at a film loading of 0.5 mg/cm^2^ and that additional film loading adds nothing to the oxidation rate. This conclusion should not depend on the specific contaminant used. The titania film of 0.74 mg/cm^2^ film loading was determined to be opaque to UVA by placing a coated plate between UV black-light lamps and a UV power meter. This finding coupled with the conclusion drawn from Jacoby’s thesis finding suggests that the UV radiation is being maximally utilized in the oxidation process. In some tests UVC radiation is used. In this case, irradiation occurs deeper into the adsorption profile for titania and similar opaqueness is anticipated due to a higher density of adsorption-allowed states.

Variation of UV intensity is achieved simply by adjusting the distance between the photocatalyst surface and lamp. Although the reactor can accommodate a photocatalyst up to 12 inches length along the flow direction, a UV opaque mask is used to select a section of the photocatalyst for irradiation. Commercial lamps of a bi-axial design are used. The lamp specifications typically includes the lamp power and flux at a selected distance from the lamp. To check for UV flux uniformity a UV meter is used to scan the exposed photocatalyst.

For the data generated by the glass-plate reactor, the oxidation rate is defined as:

r = 2.45(X_in_ − X_out_)Q/A (15)

where *r* (m-mole/cm^2^-h) is the oxidation rate, and *X*_in_ (ppmv) and *X*_out_ (ppmv), are the inlet and outlet ethylene concentrations, respectively, *Q* (lpm) is the volumetric flow rate, and *A* (cm^2^) is the area of the titania-coated glass-plate; the numerical coefficient accounts for the units change.

The absence of mass-transport effects between the photocatalyst and the flow field is demonstrated by measuring the oxidation rate as a function of the approach velocity (or volumetric flow rate) all the while keeping the residence time (length of irradiated catalyst in the flow direction divided by the approach velocity) through the reactor constant [30], Such a data plot will exhibit typically two distinct regions: a low velocity region in which the reaction rate increases with increase in the approach velocity, and a higher velocity region in which the reaction rate is constant, that is, has reached a plateau. The lowest approach velocity at which the plateau first appears established the minimum approach velocity in which mass transfer effects are negligible. This so determined minimum approach velocity is found for a specified humidity level and UV intensity. For any subsequent reaction rate that is lower than the rate at the plateau, this reaction rate is also mass-transport free. Conversely, if a change in UV flux or humidity resulted in a reaction rate greater than the one found at the plateau, then one would need to generate a new plot of reaction rate *versus* approach velocity to determine a new minimum approach velocity.

For the photocatalyst titanium dioxide, reaction rates follow Langmuir-Hinshelwood kinetics [18,20,31,32]. And for sub-ppm concentration levels the kinetics are linear dependent (see Figure 3) on the contaminant level. A large change in contaminant concentration across the photoreactor would result in a significant change in reaction rates between inlet and outlet catalyst sub-elements and the overall reaction rate is considered integral. The difference in contaminant concentration between the inlet and outlet of the photo-reactor should be kept small, a fractional change less than ~0.15 is desirable. In doing so the reaction chemistry at the reactor inlet and outlet is essentially the same and reaction rate can be considered differential. Differential reaction rates are more highly valued over integral rates as they are easier to implement in an air-purifier design code.

Humidity level has a significant impact on the reaction rate for a given contaminant [18,20,32], In these studies the reaction rate is shown to follow a bimolecular expression of the Langmuir-Hinshelwood (L-H) kinetic rate form:

(16) $$r(X_{c},X_{w},I_{G})=K_{0}\frac{K_{1}\;X_{c}}{\left(1+K_{1}\;X_{c}+K_{2}\;X_{w}\right)}\frac{K_{4}\;X_{w}}{\left(1+K_{3}\;X_{c}+K_{4}\;X_{w}\right)}$$

where *K*_0_ (m-mole/cm^2^-h) is the rate constant for a given UV intensity (*I_G_*), *K*_1_, *K*_2_, *K*_3_, *K*_4_ (ppmv^−1^) are the Langmuir adsorption equilibrium constants (ratio of adsorption to desorption rates), and *X_c_* (ppmv) and *X_w_* (ppmv) are the gas-phase concentrations of the contaminant and water vapor, respectively. The two fractional terms on the right-hand-side represent competitive adsorption between the contaminant and water for the same adsorption site [33].

As rate varies as I_a_, the value of the exponent depends on the UV level and ranges between 0 and 1 [20]. At low UV levels an exponent of 1 is expected, while at intermediate UV levels an exponent of 0.5 is expected, and at high UV levels an exponent of 0 is expected. For example, at the UV flux of 0.71 mW/cm^2^ the exponent was 0.64 for toluene, while at 33 mW/cm^2^ a value of 0.55 was found [20]. This range represents typical UV power in UTRCs PCO air purifier.

### 3.2. Modeling

As discussed, a generic photocatalytic air-purifier can be thought of as consisting, as a minimum, of three basic elements: a source of radiation, a photocatalyst and its support, and a container to support these latter two elements and as a means for channeling contaminated air from and returning treated air back to an occupied space. Commercially available cylindrical UV lamps offer an easily implemented, economical source of UV radiation. Reticulated foam and honeycomb monoliths, which have been used as supports for thermal catalysts in catalytic converters, have a long studied history, and provide suitable supports for the photocatalyst. An example diagram of a generic modular photocatalytic air purifier is displayed in Figure 1. The reactor consists of six banks of UV lamps with five photocatalytic coated honeycomb monoliths, each monolith being positioned between a banks of four lamps. Although reticulated foam and honeycomb monoliths offer similar photocatalytic performance, the honeycomb offers much lower pressure drop. Commercial and residential HVAC systems are all very sensitive to pressure drops from any ancillary environmental equipment such as air-purifiers, and so the honeycomb becomes the preferred choice for the photocatalytic reactor [32,34].

#### 3.2.1. 1-D Model

A one dimensional (1–D) model of the generic multi-stage, honeycomb-monolith photocatalytic reactor has been described in detail [13,32]. This model assumes a monochromatic radiation field at the entrance of each honeycomb channel, but allows for a non-uniform (2-D) radiation field across the honeycomb face. Within each honeycomb passage a 1-D treatment is used that incorporates gas-solid mass-transport correlations for circular flow cross-section, averaged over the channel length. Within each channel the radiation field model rigorously accounts for diffuse reflectance of the catalyst film coating on the channel walls. Reactions on the photocatalyst incorporate Langmuir-Hinshelwood kinetics to accurately account for the influence of UV flux, contaminant concentration, and humidity. 

**Figure 7:**
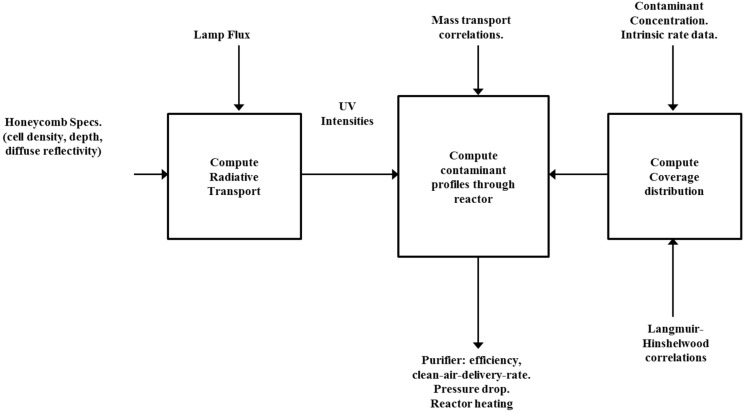
Photocatalytic reactor design flow diagram.

**Figure 8:**
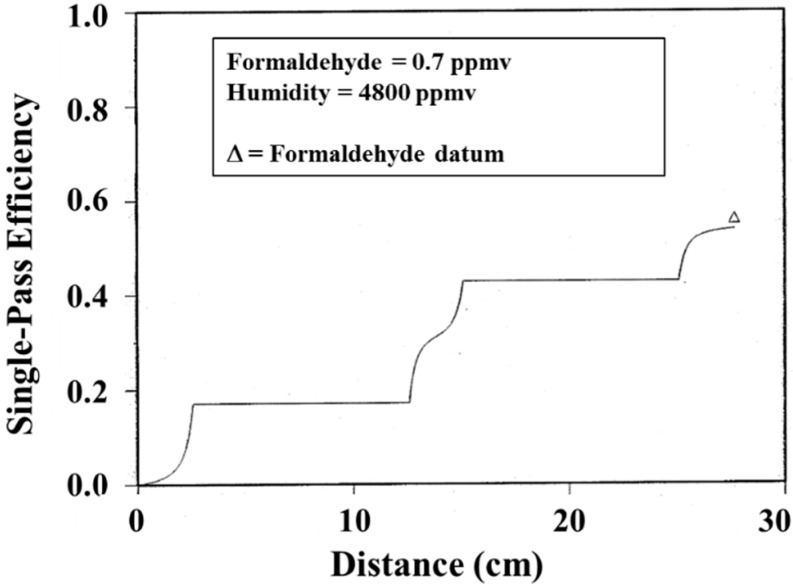
Comparison of model and experiment for formaldehyde in a 2-UV banks, 3-honeycomb monolith photoreactor. A similar version of this figure is published in Ref. [13].

A schematic diagram of the workings of the computer coded version of the model is shown in Figure 7. Because the model is essentially one-dimensional, it is fast in execution and thus makes for a highly efficient tool for engineering design.

A sample output of the model is given in Figure 8, showing the removal efficiency for the contaminant formaldehyde. In this reactor two banks of UV lamps are sandwiched between three honeycombs monoliths. A single measurement datum, taken at the reactor outlet, is included for comparison. The predicted changes in efficiency shown in the figure occur along the axial length of the honeycomb passage way. The plateaus correspond to the space between the monoliths wherein no reactions can occur. The agreement between model and the single datum is excellent.

#### 3.2.2. 3D Model

A first-principles mathematical model was developed by Hussain, *et al*., and describes the performance of a photocatalyst honeycomb monolith photocatalytic reactor for air purification [35], The single-channel, 3-D advection-diffusion-reaction model assumes steady-state operation, negligible axial dispersion, and negligible heat-of-reaction. The reactor model accounts rigorously for entrance effects arising from the developing fluid-flow field and uses a first-principles radiation-field sub-model for the UV flux profile down the monolith length. The model requires specification of intrinsic photocatalytic reaction rates that include the influence on local UV light intensity and local reactant concentration, and uses reaction-rate expressions and kinetic parameters determined independently using UTRCs flat-plate reactor [36]. Model output predictions are similar to that shown in Figure 8, and validate the 1-D model.

#### 3.2.3. 1-D and 3-D Model Validation

Both the 1-D and 3-D model predictions were favorably compared to experimental demonstration-scale formaldehyde and toluene conversion measurements for a range of inlet contaminant concentrations, air humidity levels, monolith lengths, and for various monolith/lamp-bank configurations [13,32,34]. In addition to these contaminants the 1-D model included successful comparisons with other contaminant species, such as, ammonia and hexane [32]. In the 3-D model this agreement was realized without benefit of any adjustable photocatalytic reactor model parameters, radiation-field sub-model parameters, or kinetic sub-model parameters. For formaldehyde the agreement between both models and data was excellent. Both models tended to systematically over predict toluene conversion data by about 30%, but which falls within the accepted limits of experimental kinetic parameter accuracy. The explanation for this disparity follows from the expected stronger photocatalyst bonding (affinity) for formaldehyde than for toluene [20]. As a direct consequence, photo-oxidation of toluene is more likely to be interfered by background co-contaminants. The empirical data for formaldehyde were collected at relatively high concentrations, greater than 1-ppm, while the concentration of toluene was set at a low level of ~0.3-ppm (this is necessitated by the fact that a reversible deactivation of the photocatalyst occurs for toluene levels above about 1-ppm [20], The data developed using the demonstration reactor used unfiltered building air seeded with either formaldehyde or toluene. Since background contaminant levels in the sub-ppm range would be expected, the observed systematic disparity with toluene is likely entirely or in part due to background contaminant interference. The generally satisfactory agreement between experimental and modeling data indicates that computer modeling can be used to guide the design of scaled-up reactors for practical applications. The 1-D model offers the advantage of ease of code implementation, but also is fast in execution which makes it conducive for parametric studies and as a sub-element in an optimization host code.

### 3.3. Validation

The modular design we describe has been tested in various locations, with a variety of results. Real world testing is complex, time consuming and expensive. There is no substitute for deployment of PCO based air purifiers in building environments. Laboratory conditions are well controlled environments, whereas building conditions are defined by variable source rates. Source rates vary with occupancy, usage, the age of the building and its furnishings, and with outdoor ventilation air quality. The composition of indoor air contaminates is rarely static and can vary seasonally. Given unlimited resources, a test environment (a building) could be well characterized with respect to indoor air contaminant species (both aerosol and gaseous), and with respect to hourly variations, due to occupancy and usage, and seasonal variations. A UVPCO air purifier would then be designed to act on this variable challenge and to satisfy the building HVAC design constraints. Lastly, the indoor air would be characterized again during testing with and without the use of the air purifier. In most cases compromises in the testing regimen allow validation in these environments with the resources available.

#### 3.3.1. Perceived Air Quality

Kolarick and Wargocki [37] of DTU placed a prototype air purifier, provided by UTRC, in an environment with poor air quality due to off-gassing from building materials. They showed that the air purifier had a significant effect on perceived air quality. The effect of the air purifier was to simulate an increase in e the outdoor ventilation rate by factor of 0.02 to 3. The improvement depended on the specific pollutants encountered. When the indoor air was polluted by human bio-effluents the perceived air quality worsened. This effect is attributed to an increase in byproducts from the incomplete oxidation of alcohols. Alcohols such as methanol, ethanol, amyl alcohol, *etc.* are known human bio-effluents. This effect is similar to that discussed above.

Kolarick, *et al.* [38] performed a similar experiment but included Proton-Transfer-Reaction Mass Spectroscopy (PTR-MS), Gas Chromatograhic Mass Spectroscopy and High-Pressure Liquid Chromotography measurements of trace pollutants. Analytic measurements are difficult on indoor air contaminants due to interferences between compounds and the low concentration levels and the results vary with the analytical technique used. The operation of a photocatalytic air purifier improved air quality in an office polluted by typical building materials. Of the methods used, subjective assessments and PTR-MS measurements were the most effective in demonstrating the effect. An increase in byproducts is observed by GC-MS, these byproducts are predominantly acetic acid, acetone and 2-butanone and are far below odor thresholds. These by-products do not contribute to UTRCs proposed tolerance metric as they are limited by odor threshold. PTR-MS did not show the same increase, but detected a few ppb increase in formaldehyde, acetaldehyde and acetone during the first hour after start up. These species will contribute to the tolerance metric and these byproducts must be well understood to determine any impact on human health.

Both the above studies were performed with a high efficiency (seven stages) UVPCO prototype air purifier, provided by UTRC, and the conclusions pertain to perceived air quality, not occupant health.

#### 3.3.2. Analytical Air Quality

In a similar study, a smaller prototype (two stages) air purifier was supplied to LBNL, who challenged the reactor with a mixture of 27 VOCs commonly found in office buildings. Hodgson, *et al.* [22] reported that operation of the air purifier compensated for reducing the outdoor air intake by 50%. They assumed the unit was installed in the building air flow after the outdoor and recirculated air are combined and the recirculated flow was three times the outdoor air intake. In addition they observed formaldehyde, acetaldehyde, acetone formic acid and acetic acid byproducts. Of these, formaldehyde would contribute the most to an air quality metric such as the tolerance index.

#### 3.3.3. Building Air Quality

**Figure 9:**
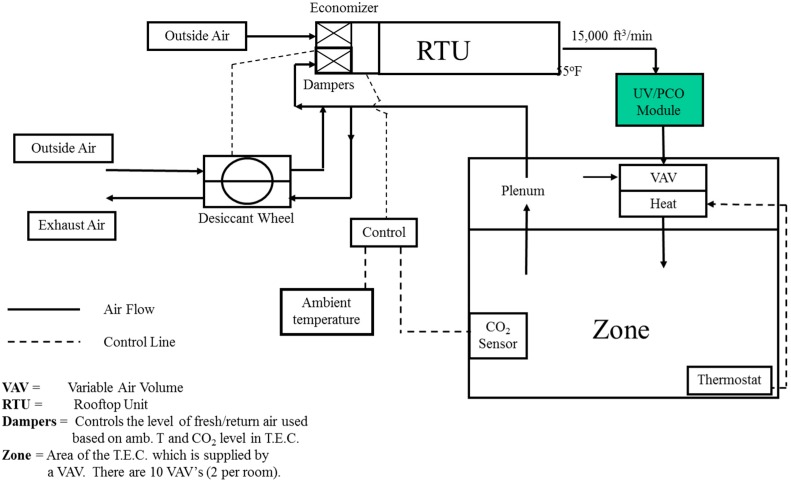
HVAC Schematics of air purifier installation; 35 ton rooftop with VAV, economizer and desiccant wheel.

Lemcoff, *et al.* [39] performed a building study at UTRC’s Technical Education Center where a specifically designed UVPCO air purifier is installed in a rooftop unit that feeds a *ca*. 100 person capacity educational room. VOC testing is carried out using US EPA protocol techniques both prior to and during air purifier operation. Summa canisters are used to sample hydrocarbons and chlorinated solvents and adsorbent cartridges to analyze for aldehydes. The application of the URC proposed Tolerance Index is used to determine the level of air quality. The purifier is installed in the recycled air (nominally 15,000 ft^2^/min, 55 °C) subsequent to the 35 ton Rooftop Unit and prior to variable air ventilation (VAV) unit at the room exhausts. An economizer was installed to lower the rate of outdoor ventilation in good weather and humidity was controlled through use of a desiccant wheel. A schematic is shown in Figure 9. Sensors are used to continuously monitor temperature, relative humidity, particle concentration, total VOC and CO_2_. Comparable pollutant levels to those found in the EPA BASE study are found. When UTRCCo proposed tolerance metric is applied to the levels found for one study performed with an occupied room (50 occupants), operation of the air purifier is predicted to reduce the tolerance metric from a steady state of *ca*. 0.64 without air purification to a steady state of 0.29 with the air purifier. The outdoor air flow was reduced during operation to show potential energy savings. Application of the tolerance metric to the indoor air with purification and with reduced air show the Index varied over the range 0.48 to 0.62, indicating that air quality was maintained with reduced air flow and 15% energy savings. The reduced flow would also allow for a 17% capacity reduction in the roof top unit. These results are consistent with those of LBNL. Unfortunately, when the catalyst is examined after three months of operation the catalyst was found to be partially deactivated. Some catalyst activity was regenerated by operation in a clean humid environment for several days.

UVPCO air purifiers deployed in both the US and France [6] were tested for catalyst efficiency after varied periods of operation in different office environments. Catalyst deactivation is observed in all cases, some deactivation was reversed when catalyst monoliths are exposed to UVC operation in a clean environment. The catalyst used was either 100% Degussa P-25 or a mixture of 50% Degussa P-25 and 50% Millenium 50 supported on aircraft grade aluminum honeycomb. Examination of catalyst surface by XPS shows a surface contaminated with silicon, carbon and to a lesser extent nitrogen. Electron microprobe data of a catalyst taken from an air purifier deployed in France indicated a strong 230–260 nm Si rich layer on the surface of the catalyst. Silcones are the most prevalent silicon containing VOC or SVOC components of indoor air. Hay, *et al.* [6] studied siloxane deactivation of titania in the flat plate reactor described above. A strong correlation is observed between surface Si as seen by XPS and the extent of deactivation. They also show a strong correlation of the extent of deactivation observed in catalysts (3% WO_3_ on Degussa P-25) deployed in prototypes operating in office environments, with the laboratory results. They attribute purifier deactivation to ambient siloxanes oxidized by PCO action to amorphous SiO_2_. This is observed by TEM in catalysts 80% deactivated by PCO in the presence of tetramethyl siloxane (TMS or D4 siloxane). Ambient air testing in the locations where the prototypes were deployed indicated an average siloxane concentration of 0.2 ppmv was present with DMCPS (decamethylcyclopentasiloxane) the most prevalent. Hay, *et al*., also demonstrate that UVPCO air purifier lifetime can be extended with an adsorbent prefilter selected to adsorb high molecular weight compounds such as siloxanes.

Validation of UVPCO air purifiers in laboratory situations simulating indoor air and in buildings, where prototypes operated in an actual office environment yield results that are both promising and disconcerting. These air purifiers can impact the quality of air positively but questions remain about the potential impact of byproducts and the lifetime of catalysts. In order to provide impetus for the deployment of this technology as product, consumers must see a tangible benefit. Improved air quality has been linked to improved productivity, but no metric exists correlating measurable quantities directly to improved air quality. Building owners, concerned with the cost of day-to-day operation of building HVAC systems see no payback in nebulous increased productivity but look to the hard numbers available for energy use (operating cost) and equipment capacity (capital cost).

The demonstration conducted in UTRC’s Technical Education Center indicates that both capacity reduction and energy use reduction can be obtained utilizing UVPCO air purification to reduce outdoor air intake. This is validated by the work done at DTU and LBNL. This indicates that a business case can exist to replace outdoor air with purified air. UTRC’s proposed tolerance metric, if valid, is a strong indicator that this can be done while simultaneously improving total air quality. At this stage of development, however, each case must be considered independently. This business case depends strongly on ambient air intake, if little cooling or heating is required, there are little energy savings to be gained by reducing the extent of conditioning. In addition, the effects of byproduct formation must be better understood in terms of their impact on human health.

### 3.4. Current and Future Research

As we have discussed, ambient air itself is an elusive target, chemical off-gassing changes as new materials are developed and incorporated into building materials, furnishings, clothing, and personal care products. A century ago no concerns over siloxanes existed, now silicon oxide whiskers grow on electrostatic particle removers, interfering with operation [40], and causing UVPCO deactivation. Siloxanes appear to be ubiquitous and have even been identified in Artic ice [41].

The action of a photocatalytic air purifier can change ambient air. A purifier can mineralize VOCs if designed and operated appropriately, but is cost prohibitive to do so. One hundred percent mineralization is achievable only in a laboratory. Spatial requirements in buildings dictate the use of small air purifiers. These have various single pass efficiencies which depend, as we have discussed, on species, concentration, temperature and coadsorption phenomenon. In addition, they depend strongly on the choice of catalyst. BET surface area, porosity, crystallinity and surface morphology can affect adsorption. In addition the photocatalyst surface changes over time as various types of deactivation occur. As deactivation reduces the number of active sites available, incomplete mineralization becomes more prevalent, promoting the production of byproducts. Not only is by-product formation a concern when the catalyst surface is pristine, but the effect of deactivation on byproduct formation must be evaluated. As active surface sites are removed from photocatalytic action, the numbers of radicals available for mineralization decrease. This decrease may simulate the effect of increasing concentration. In other words, as the ratio of available radicals to parent molecules is decreased, complete mineralization is decreased.

To facilitate commercialization of this technology, a critical need exists to minimize the effect of deactivation and to increase photoreactivity. Extending the wavelength region for efficiency photon adsorption further into the visible can also increase the ease of commercialization. The business case in buildings is based on the capital cost of the air purifier and the cost of operation. Is there a return on investment? The efficiency of contaminant removal relates directly to the size of the air purifier and therefore the capital cost. The catalyst lifetime relates directly to the operating cost. If the lifetime is too short the return on investment time increases. Extension of excitation wavelength into the visible could allow the use of LEDs, which are commercially available at longer lifetimes in longer wavelengths. This in turn could lower both capital and operational costs due to the choice of light source. Current activity ongoing at the University of Connecticut is focused on efficient siloxane trapping, deactivation resistant photocatalysts, high efficiency catalysts and visible light activated catalysts.

As was described previously, deactivation of metal-based catalyst through either coking or poisoning by particular species has been a continuing problem. One such example is that of the titanium dioxide system, in which TiO_2_ is deactivated via siloxane groups that deposit amorphous SiO_2_ onto the surface of the catalyst. The main effect of this is partial to complete deactivation of the catalyst, dependent on the amount of siloxane present [6]. This occurs through the blocking of active sites (and the surface) by the silica deposited, eventually leading to almost total deactivation.

To counteract deactivation, a variety of methods have been employed, including the development of a high surface area material and the tuning of the surface area and pore sizes of the material. The objective is to create an active material where deposits of amorphous silica do not encapsulate the surface. UTRC patented one such a technique [42], and another was pioneered at UConn by Horvath [43]. Through heat treatments, these parameters can be tuned. Additional research has shown that pore size and surface area can be maintained at high calcination temperatures through the use of silane coatings followed by the removal of the silica layers post heat treatment. A porous silica overlayer can extend catalyst lifetime [44,45].

**Figure 10:**
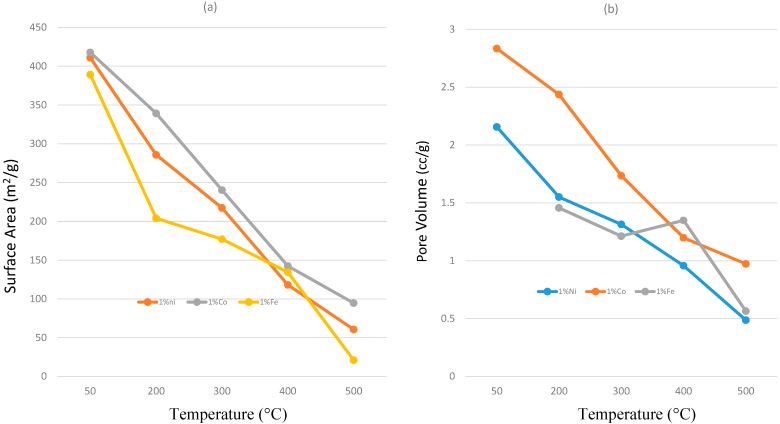
(**a**) Surface area change and (**b**) pore volume change in doped titania systems as a function of calcination temperature (T = 50 °C, 200 °C, 300 °C, 400 °C and 500 °C).

Figure 10 shows the dependence of surface area and pore volume on temperature for doped titania systems. Increases in temperature lead to decreases for both. From these images, these parameters may be modulated via different dopants, though studies revolving around these materials are ongoing. Increases in overall surface area and pore volume are theorized to increase the lifetime of the catalyst system as the loading of silica required to deactivate would theoretically increase.

One attractive technique is to simulate the high activity of Degussa P25 in a high surface area material. P25 is a benchmark material for titanium based photocatalyst studies, employed by UTRC and many other laboratories. Since 1990, significant research has been focused on P25 due to its unique composition and high relative activity. This high activity has been shown in different photocatalytic reaction systems but few are better photocatalytically under similar conditions. The reason why P25 has such excellent activity is attributed to a synergetic effect between anatase and rutile [46]. P25 is a mixed phase material that is composed of anatase and rutile phases, the specific ratio has been reported between 80:20 and 75:25 [47]. While P25 is attractive photocatalytically, its disadvantages are not negligible. The surface area of P25 is relatively low (around 50 m^2^/g). P25 is a nonporous material and is visible light inactive. After intense studies on P25, researchers are trying to develop new photocatalysts that have P25hat haveactivity combined with a high surface area, a porous structure and are visible light active.

To achieve high surface area with a porous structure, mesoporous TiO_2_ materials are a prime candidate. Mesoporous TiO_2_ was first synthesized by Antonelli *et al.* in 1995 [48]. The reported surface area of mesoporous TiO_2_ material is as high as 1200 m^2^/g [49]. This high surface area of offers more active sites during photocatalytic reactions. Moreover, the nanocrystalline walls and the pore structure will allow photogenerated electrons and holes to reach the surface easier and further favor the surface photocatalytic oxidation/reduction reactions. The main drawback of mesoporous TiO_2_ materials is their low thermal stability. Thus, most of the mesoporous TiO_2_ materials can only possess the anatase phase. Mixed phase or rutile phase titania materials are difficult to form while maintaining the mesopore structure.

To achieve visible light photocatalytic activity, metal and non-metal dopants are commonly used to stretch the absorption band of photocatalysts to the visible light region. Different metals have been doped into TiO_2_ nanomaterials; W, V, Ce, Zr, Fe, Cu, Ag are all shown to effectively stretch the absorption band to the visible light range (>400 nm) [50,51]. Not only metal dopants, but also various nonmetal elements such as B, C, N, F, S, Cl and Br have been successfully doped into TiO_2_ nanomaterials. After dopants have been introduced into the TiO_2_ structure, the electronic, optical and photoelectrical properties of TiO_2_ photocatalyst are modified [52].

One of the important properties of dopant modified TiO_2_ structures is the phase. The most photocatalytic active phase is the anatase phase, which has a band gap energy at 3.2 eV (387 nm). The thermally stable phase is the rutile phase, which has a band gap energy of 3.0 eV (412 nm). The anatase phase is not visible light active. The photon energy from visible light (>400 nm) is not strong enough to separate electron/hole pairs. Theoretically, the rutile phase should be photocatalytically active under visible light with an appropriate band gap. However, rutile shows a high recombination rate which leads to poor photocatalytic activity. Combining to make a mixed phase material should lead to photocatalytic activity that should be significantly improved. Commercially available P25 is the best example of such mixed phase materials.

If one takes advantage of a mesoporous structure and the mixed phase simultaneously, the photocatalytic activity of this material should be significantly improved. Achieving two phases (anatase and rutile) and maintaining a mesoporous structure at the same time is the biggest challenge. Phase transformation promoters could significantly decrease the anatase-to-rutile phase transition temperature. Al, Co, Cu, Fe, Cr, V and Zn are all reported as efficient phase transformation promoters [53,54,55,56,57,58]. If the transition temperature can be brought to lower than lower than 450 °C, a mesostructure can be maintained at the same time.

A mesoporous mixed phase TiO_2_ material is expected to be an efficient visible-light-activated photocatalyst. The mesopores facilitate the diffusion of photogenerated electrons and holes to the particle surface. More importantly, the synergetic effect between the anatase phase and the rutile phase causes an efficient charge separation across phase junctions. The mesoporous mixed phase TiO_2_ will not only have enhanced photocatalytic activity but also higher adsorption ability, improved thermal stability, longer life and be suitable for both liquid phase and gas phase reactions.

Recently developed University of Connecticut (UCT) mesoporous materials offer the opportunity to prepare mesoporous mixed phase TiO_2_ materials [59]. Through controlling the sol-gel chemistry of inorganic sols in inverse micelles and NO_x_ chemistry, highly thermal stable highly thermal stable (450 °C) mesoporous TiO_2_ materials can be prepared. Further, by modifying the synthesis method with certain metal dopants (phase transition promoters), thermally stable mesoporous TiO_2_ with mixed phase can be prepared.

**Figure 11:**
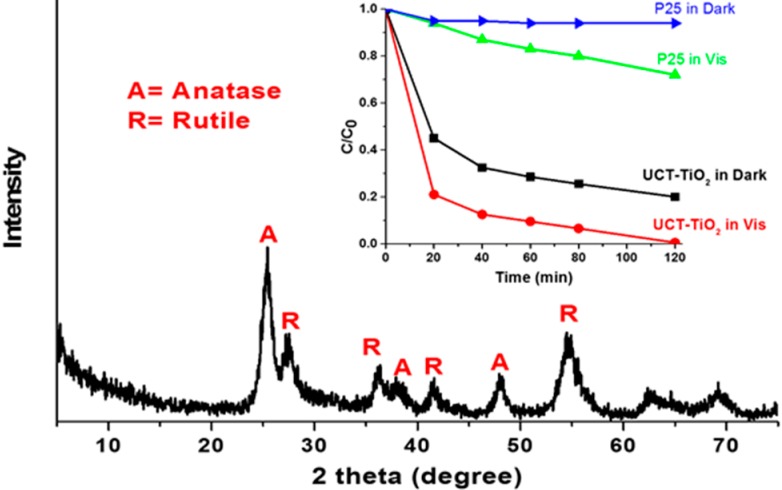
X-ray diffraction pattern for UCT-TiO_2_ materials. Inset image: adsorption ability and photocatalytic ability of UCT-TiO_2_ compared to P25 tested in dark and visible light conditions, respectively. The efficiency was calculated by C/C_0_ (dye concentration in different time/initial dye concentration).

The mesoporous mixed phase TiO_2_ (UCT-TiO_2_) was prepared based on the method discussed above. This material is composed of 60% anatase and 40% rutile based on calculations from X-ray diffraction patterns. The adsorption ability of UCT-TiO_2_ was compared with Degussa P25 in the dark to adsorb methylene blue (MB) dye in 2 h. Figure 11 shows that the UCT-TiO_2_ shows much higher adsorption ability than P25. 

In addition, the visible light (>400 nm) photocatalytic activity of UCT-TiO_2_ was tested by degrading methylene blue dye. As shown in Figure 10, MB dye can be completely removed by UCT-TiO_2_ in 2 h. P25 displays poor degradation performance under visible light.

The mesoporous mixed phase TiO_2_ (UCT-TiO_2_) is a potential new photocatalyst that can be used in liquid phase reactions such as dye degradation, organic compound decomposition, and also for gas phase reactions such as VOC degradation and CO oxidation reactions. The unique mesostructure combined with the synergetic effect between mixed phase junctions offers the opportunity for more potential photocatalytic applications. This type of catalyst is promising for increasing reactor performance for indoor air, and potentially allowing energy efficient LED activation.

Siloxanes are a class of anthropogenic chemicals having a multitude of applications in the production of household, automotive, construction, and personal care products, as well as acting as intermediates in the production of silicon polymers. Siloxanes are found to be ubiquitous in the air, water, sediment, sludge, and biota. Due to their widespread use, siloxanes have received notable attention as emerging organic environmental contaminants over the past two decades. Most low molecular weight siloxane compounds volatize quickly into atmosphere to pollute the air and high molecular weight siloxane compounds remain in the water and soil. Siloxanes need to be cleaned in the air because of their potential for long range transport and bioaccumulation. They also, of course, deactivate photocatalysts. Table 3 lists common types of linear and cyclic siloxanes.

**Table 3:** Siloxanes and their physical properties.

Siloxane Type	Formula	Abbreviation	Molecular Weight	Vapor Pressure (Torr, 25 °C)
Hexamethyldisiloxane	C_6_H_18_OSi_2_	L2, MM	162	31
Octamethyltrisiloxane	C_8_H_24_O_2_Si_3_	L3, MDM	237	3.9
Decamethyltetrasiloxane	C_10_H_30_O_3_Si_4_	L4, MD_2_M	311	0.43
Dodecamethylpentasiloxane	C_12_H_36_O_4_Si_5_	L5, MD_3_M	385	0. 1022
hexamethylcyclotrisiloxane	C_6_H_18_O_3_Si_3_	D3	222	10
Octamethycyclotetrasiloxane	C_8_H_24_O_4_Si_4_	D4	296	1.3
Decamethylcyclopentasiloxane	C_10_H_30_O_5_Si_5_	D5	371	0.4
Dodecamethylcyclohexasiloxane	C_12_H_36_O_6_Si_6_	D6	445	0. 0494

As we have seen, siloxanes in ambient air can disrupt the operation of a PCO air purifier causing rapid deactivation through conversion to amorphous silica on the catalyst surface. Hay, *et al.* [6] demonstrated that approximate lifetime doubling occurs when an air purifier is protected by an adsorbent filter. Lifetimes can be extended further as more efficient siloxane traps are developed. There are various methods to remove siloxane including biological methods, cooling, absorption, catalysts and adsorption. Among these techniques, solid adsorbent is the simplest way to remove siloxane for which various types of active materials have been applied such as silica gel, alumina, activated carbon and so on (Table 4). The pollutant is adsorbed by physical interaction with the surface.

**Table 4:** Siloxane adsorbents in the literature.

Type of Materials	Materials Details	Adsorption Capacity (g Siloxane/g Adsorbent)	Surface Area (m^2^/g)	Type of Siloxane	Ref
Molecular sieve	13× molecular sieve 45/60 mesh	0.01	-	D5	[59]
Zeolite	Faujasite NaX	0.276	500	D3	[60]
activated carbon (ACs)	NORIT RB4	0.41	>1000	D3	[61]
MgO	Commercial, periclase phase	N/A	31	D3	[60]
CaO	Ex-CaCO_3_ calcination, cubic phase	0.003	31–40	D3	[60]
Silica gel	Fluka (particle size: 1–3 mm)	0.1	-	D5	[59]
Alumina	Duksan Co. (65.7% porosity)	0.1775	-	D4,D5	[62]

At present, active carbon is the most efficient and cheapest solid adsorbent which is used to clean the siloxanes in the air.

However, active carbon will have a dramatic decrease of performance in the presence of humidity. This is attributed to blocking the adsorption sites by the formation of hydrogen bonds [63]. Mesoporous aluminosilicate (UCT-15) belongs to the family of UCT mesoporous materials, which have high surface area, crystalline walls and monomodal pore size distributions. The UCT materials have been used for H_2_S adsorption and showed remarkably high adsorption capacities.

The mesoporous aluminosilicate was synthesized by dissolving TEOS and aluminum nitrate (Si:Al molar ratio = 5) in a solution containing HNO_3_, 1-butanol, and P123 in the beaker at room temperature and under magnetic stirring. The obtained clear gel was placed in oven for 4 h. Then samples were grained and calcined under air at 450 °C for 4 h (2 °C/min heating rate). 

Adsorbent performance of D4 adsorption tests were run by passing adsorbents with carrier gas (N_2_) which contains siloxane and water moisture. The concentration of water moisture in the carrier gas was 1.7% (molar). After passing through the adsorbents, the gas wash bottle was used to adsorb the residue siloxane in the carrier gas. GC/MS with a DB-5 column was used determine the siloxane concentration in the trap solvent.

**Figure 12:**
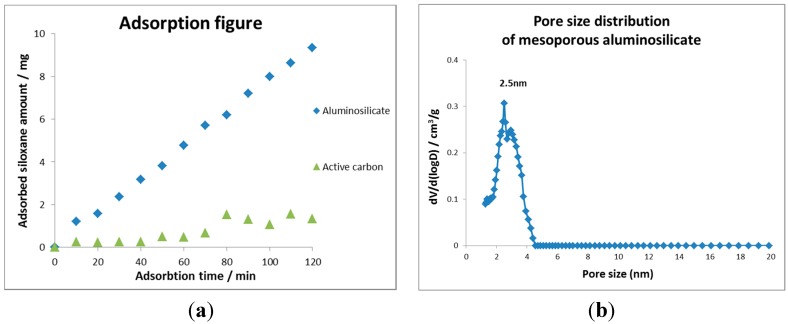
(**a**) Adsorption of siloxane on the adsorbents over time, UCT material compared to activated carbon; (**b**) Pore size distribution of mesoporous aluminosilicate.

Figure 12a shows adsorbed amount of siloxane over time by mesoporous aluminosilicate compared with active carbon. On the adsorption figure, the more siloxane the adsorbent adsorbed, the better the adsorbent. Figure 12b shows the pore size distribution of mesoporous aluminosilicate adsorbent and the surface area of aluminosilicate is 229 m^2^/g. The pore size distribution figure confirms the mesoporous structure of mesoporous carbon adsorbents. We can see from Figure 12a that mesoporous aluminosilicate works much better than actived carbon under the moisture condition. This is because the hydrophobicity [64] of the aluminosilicate surface could weaken the blocking effect of active adsorption sites.

Manganese oxides comprise a large group of compounds due to the multi-valent nature of such oxides and the complexity of the forms in how the octahedral units connect with each other. The application of these compounds has been pursued widely in adsorption, catalysis, energy storage and environmental pollutant removal [65]. In particular, due to the strong oxidative properties, manganese oxide was successfully used for abatement of a large category of environmentally hazardous materials. These include carbon monoxide [66,67], VOCs [68], dyes [69], hydrocarbons [70], halogenated hydrocarbons [71], and organophosphates [72]. Both thermal and photocatalytic processes were studied to achieve sizable decomposition of the pollutants [70,73]. The photocatalytic approach is beneficial for consuming less energy when conducted under ambient conditions as compared to the harsh conditions of thermal degradation. Amorphous manganese oxide (AMO) shows remarkable activities and stabilities in oxidative photocatalysis [70] and is a promising candidate for titania replacement in a PCO air purifier. A typical synthesis of AMO is done by reducing KMnO_4_ with oxalic acid under ambient conditions [74].

**Figure 13:**
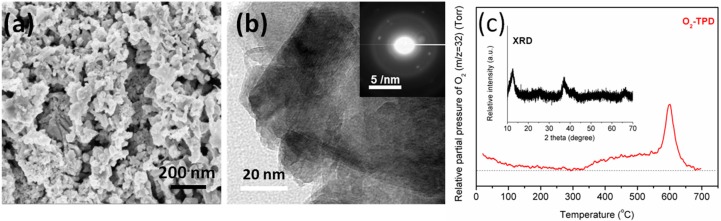
(a) SEM images; (**b**) HRTEM, (in set, selected area diffraction (SAD)); (**c**) O_2_-temperature programmed desorption (inset, XRD pattern) of AMO.

Figure 13a,b show the morphology of AMO. The amorphous nature can be seen from both the weak X-ray diffraction (Figure 12c inset) and high resolution transmission electron microcopy (HRTEM) (Figure 13b) data. Figure 13c displays the O_2_-TPD profile of AMO, which exhibits two desorption peaks. The first broad peak that spans over 350–550 °C represents the large amount of adsorbed oxygen; the second one centered at 600 °C indicates lattice oxygen [74]. 

The uniqueness of AMO is due to the composition of randomly oriented nanosize domains (*ca*. 10 nm), mixed valency, and large surface area (*ca*. 180 cm^2^/g). More importantly, the good photocatalytic activity was correlated with the high mobility of lattice oxygen and ample surface adsorbed oxygen, and their migration to and from atmospheric oxygen, which assures high stabilities under continuous irradiation [75]. The transformation of surface adsorbed oxygen upon irradiation can be described by the following equation [75]:

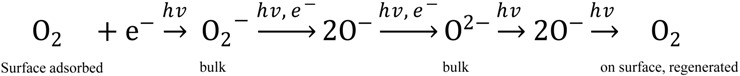
(17)


A plausible explanation of the photo activity is that surface adsorbed O_2_ can accept photogenerated electrons, and become O_2_^−^. Upton further excitation under light, O_2_^−^ reacts with electrons and forms oxygen radicals (O^−^), which continue to react with electrons to form O^2−^(bulk). Next, O^2−^(bulk) could migrate to the surface as O_2_ (regenerated). In the whole process, radicals such as **^·^**OH and O_2_^−^ (superoxide anion radicals) could be formed and play reactive roles. Both species were probed in a recent study utilizing AMO as a photocatalyst to degrade *N*- nitrosodimethalamine (NDMA) to form NO_3_^−^ and HCOH. The efficiency of the degradation is comparable to that of TiO_2_ (Degussa P25) as a photocatalyst [75].

Considerable work has been done toward liquid phase dye degradation with different manganese oxide catalysts in our group. Segal *et al*. [76] use octahedral molecular sieves (OMS), octahedral layered (OL), and amorphous manganese oxide (AMO) materials to decompose pinacyanol chloride (PC) dye. The result shows that metal-doped OMS-2 materials have the highest activities compared with OMS-1, OL-1, and commercial MnO_2_ in the rate of decomposition of PC dye. The presence of H_2_O_2_ can inhibit the dye degradation ability of the manganese oxide materials. Other parameters such as catalyst concentration, pH, and structural changes that occur in the catalysts are also studied in the literature. The manganese oxide catalyzed dye decomposition follows an adsorption/oxidation/desorption process. Sriskandakumar *et al.* [69] reported decomposition of methylene blue (MB) dye with several doped and undoped OMS manganese oxide materials by using tertiary-butyl hydrogen peroxide (TBHP) as oxidant which could enhance the degree of MB dye decomposition.

**Figure 14:**
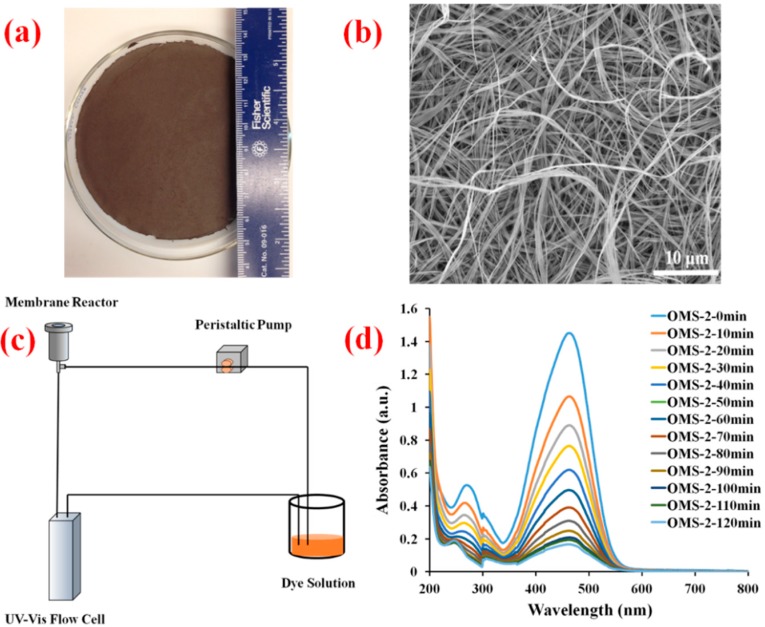
MnO_2_ nanowire membrane for dye degradation: (**a**) overall view of MnO_2_ nanowire membrane; (**b**) SEM image of the MnO_2_ nanowire membrane; (**c**) *in-situ* membrane reaction system; (**d**) the UV-Vis spectra of the catalytic degradation of methyl orange by MnO_2_ nanowire membrane.

Figure 14 shows the free-standing membrane from the self-assembly of ultralong MnO_2_ nanowires recently made by our group for an *in-situ* dye degradation study. The smooth MnO_2_ nanowire membrane (Figure 14a) was made according to Yuan’s method [77]. The nanowires (Figure 14b were uniformly distributed with lengths of tens of micrometers. With the *in-situ* membrane reaction system (Figure 14c) the dye degradation reaction can easily be controlled. Methyl orange dye was partly decomposed after 120 min at room temperature by MnO_2_ nanowire membrane (Figure 14d). Further studies are still going on with different metal doped MnO_2_ nanowire membranes and reduced graphene oxide (RGO)/MnO_2_ nanowire membranes for different organic dye decompositions.

Developing these various materials can enable cost effective, efficient deployment of photocatalytic air purifiers.

## 4. Conclusions

Understanding the effect of a photocatalytic purifier on air is best accomplished by thorough study of the coadsorption phenomenon. In a simple system with limited quantity of contaminants the study is straightforward. The PCO reactor can be designed to be cost effective and practical. In indoor air the system is complex and varying, the design of the air purifier is constrained by cost and the assumptions made about the environment. Practical validation experiments have demonstrated that two significant barriers remain, catalyst lifetime and by-product formation.

Photocatalyst deactivation has been demonstrated to occur rapidly in ambient air containing siloxanes. Sequential radical attack on the parent molecule creates an amorphous silica cap on the catalyst surface preventing further oxidation of air entrained contaminates.

Mineralization by-products occur due to incomplete oxidation of parent VOCs. When the number of active sites is large compared to the adsorbed species, mineralization is encouraged and by-products are minimized. This is seen in product space maps such as the example shown with TCE. Conversely, when surface coverage of the parent molecule is high, this ratio decreases and incomplete mineralization occurs. This is seen both at high TCE concentration and in PCO purifier validation studies when alcohols are present in the ambient airstream. If this ratio impacts complete mineralization and byproduct formation, then the effect of an ageing catalyst also could be significant. As the PCO air purifier operates in ambient air, deactivation occurs and byproduct formation could increase with the decreasing number of active sites.

Of these effects, rapid deactivation is the most significant impediment to implementation of this technology. Pre-removal of siloxane or other deactivating agents and/or the development of deactivation resistant catalysts is critical. Higher surface area catalyst with an increased number of active sites could decrease the formation of byproducts. Visible light photocatalysts could impact operational costs. All these areas are fruitful avenues for further study, providing ample grist for the academic mill.

PCO air purifiers are viable now for simple systems, such as remediation or industrial waste streams. They are not viable for indoor air until the effect by-product formation is understood and minimized and catalyst lifetime is extended.

Other research groups have contributed significantly to the application of photocatalytic technology to indoor air. Other reactor designs will affect the parameters we have discussed, some modifying the effect of byproduct formation through longer dwelling time. Each modification to ameliorate one problem may exacerbate another. Longer dwelling time may increase pressure drop increasing cost of implementation and may promote deactivation by siloxanes. The authors have attempted to describe through reviews the methodology appropriate to photocatalytic product development; and how to design, prototype, and validate those products in the real world. There are of course a wide variety of reactor designs that offer a different set of barriers to product development. Many of these designs have been proposed and tested in academic environments but few, if any, have been developed into products and tested in buildings. It is the same methodology described in this review that is appropriate for product development based on current and future reactor innovations. It is the manner in which UTRC approached this very complex and intriguing problem and the knowledge gained during this journey that is instructive for future product development.
